# Convergent Double Coronary Sinus Potentials During Atrial Tachycardia

**DOI:** 10.19102/icrm.2023.14041

**Published:** 2023-04-15

**Authors:** Idriz Merovci, Idris Yakut, Oktay Gulcu, Abdullah Tuncez, Meryem Kara, Elif Hande Ozcan Cetin, Ahmet Korkmaz, Ozcan Ozeke, Serkan Cay, Firat Ozcan, Dursun Aras, Serkan Topaloglu

**Affiliations:** ^1^Department of Cardiology, University Clinical Center of Kosovo, Prishtina, Kosovo; ^2^Department of Cardiology, Health Sciences University, Erzurum Education and Research Hospital, Erzurum, Turkey; ^3^Department of Cardiology, Selcuk University, Konya, Turkey; ^4^Department of Cardiology, University of Health Sciences, Ankara City Hospital, Ankara, Turkey; ^5^Department of Cardiology, Istanbul Medipol University, Istanbul, Turkey

**Keywords:** Atrial electrogram, atrial tachycardia, coronary sinus

## Abstract

The analysis of the patterns and timing of coronary sinus activation provides a rapid stratification of the most likely macro–re-entrant atrial tachycardias and points toward the likely origin of centrifugal ones by comparing the left atrial and coronary sinus activation sequence and morphology during sinus rhythm and atrial tachycardia. The analysis of both the near- and far-field electrogram morphology of atrial signals also gives important clues in determining the mechanism of the arrhythmia.

## Case presentation

**Figure 1:**
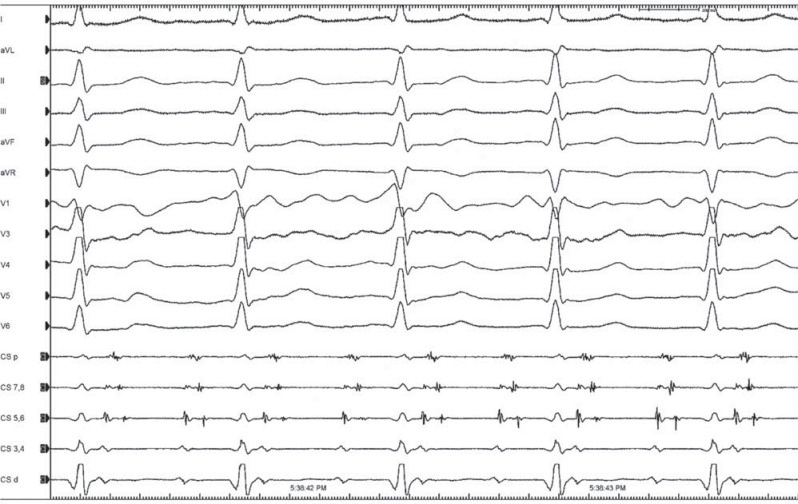
Coronary sinus electrograms showing the atrial tachycardia with a 2:1 atrioventricular conduction at a cycle length of 260 ms.

A 64-year-old man who had undergone mitral valve replacement via median sternotomy 11 years earlier was referred for catheter ablation of symptomatic left atrial (LA) tachycardia (AT). He had undergone conventional mitral isthmus (MI) ablation, with connection of the mitral annulus and ostium of the left inferior pulmonary vein, 9 years ago.^1^ A baseline electrocardiogram (EGM) revealed AT with a 2:1 atrioventricular conduction at a cycle length of 260 ms. The decapolar diagnostic catheter was placed in the coronary sinus (CS), and double atrial electrograms with convergent activation wavefronts were recorded during the left AT **(Figure 1)**. What is the mechanism of these convergent double potentials (DPs) during atrial tachycardia?

## Discussion

Until recently, most clinical electrophysiologists believed that CS electrograms strictly reflect the LA activation.^2^ Indeed, the CS constitutes the fifth chamber of the heart, and the recordings from catheters within the CS have near-field (NF) CS muscle signals. However, the recognition of far-field (FF) LA potentials distinct from the NF potentials of the CS muscle has increased after some detailed human anatomical and histopathological studies revealed a distinct spatial relationship and muscular connections between the CS muscularity and the LA myocardium.^3^ The variability of the conduction through the CS–LA connections, related to their intrinsic conductive properties (conduction delay), might lead to a variable relationship between the endocardial and the epicardial activation.^4–6^ Therefore, the CS atrial potentials recorded by an electrode catheter placed in the CS generally consist of the DP that represents both the dull FF component from the contiguous LA myocardium and the sharp local NF component from CS musculature.^3,5,7,8^ In the vast majority of the cases, the CS electrograms have a dull-sharp sequence.^9^ Although the local NF CS signal is usually used as a surrogate for the adjacent LA FF signals during mapping from the CS,^10^ careful signal analysis and discrimination of the local NF CS from the FF-LA potentials might be necessary for some complex situations.^2^ During ablation of left-sided atrioventricular re-entrant tachycardias, the separated DP of the atrial electrograms in the CS may suggest the connection of the MI inside the CS (epicardial connection) despite the conduction block in the LA (endocardial conduction block).^11–14^ While the sharp potentials (NF components of the CS) demonstrate persistent conduction through the MI, the dull potentials of the FF component indicate the conduction block of the MI in the LA.^15^ Beyond the intra-atrial MI conduction block during ablation of the left lateral accessory pathway, detailed characterization of the complex forms of AT also relies on the correct interpretation of intra-atrial electrograms, comparing the LA and CS activation sequence and morphology during sinus rhythm and AT.^8^ Most often, DPs represent sequential activation of tissue on each side of an anatomic or functional barrier of a re-entrant circuit. Under such circumstances, they are recorded in the center of the circuit. Alternatively, DPs may reflect sequential activation of tissue on each side of a conduction barrier where the impulse passes only through a narrow isthmus with conduction delay.^16^ Therefore, the differential diagnosis of sequential activation of the LA and CS musculature includes AT (1) incorporating both the LA and CS musculature as essential components of the re-entrant circuit^17^; (2) with the LA and the CS musculature both passively activated as bystanders^18^; or (3) with the LA as an essential component of the circuit and the CS musculature as a bystander pathway, including AT circulating around the mitral annulus (peri-mitral AT).^19,20^ Herein, the line of the block (LOB), characterized by distinct electrograms separated by an isoelectric line, is associated with wavefront collusion (WFC) and is not expected to produce complex electrograms, as the wavefronts do not cross these lines but instead arrive at different times, producing the classic atrial DPs separated by an isoelectric line.^21^ In general, the WFC usually displays normal bipolar voltages, suggesting the absence of myocardial fibrosis or scar.^21^ It is therefore linked to a functional and dynamic mechanism rather than a substrate and is characterized by a short-duration, single-component EGM.^21^

**Figure 2:**
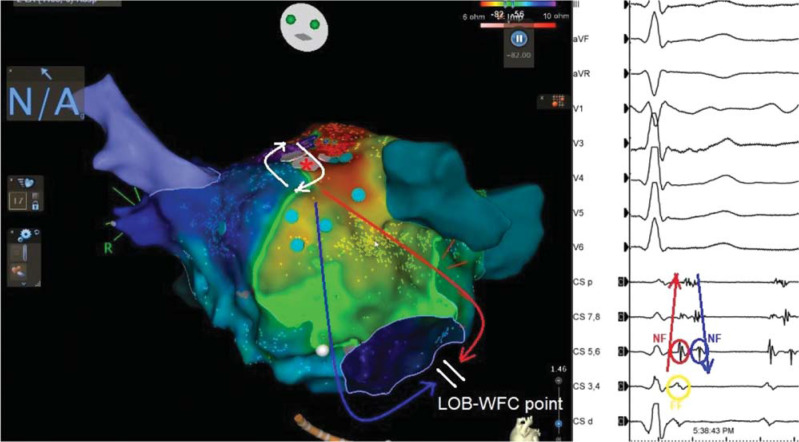
Activation mapping shows the re-entry circuit at the anteroseptal left atrium (white circuit). The red asterisk shows the successful ablation point. The line of the block and the collision of the wavefront collision point are seen on the mid-distal coronary sinus (CS) as compatible with double near-field potentials (blue and red arrows and circles) within the CS. The yellow circle shows the far-field left atrial signal within the CS.

In the current case, the patient had a history of previous MI lines for left AT. Careful examination of these DPs within the CS revealed a pair of opposing activation wavefronts with sharp and high-frequency NF signals (**Figure 2**, blue and red ones), indicating the local NF CS signals, suggesting the LOB, which makes peri-mitral re-entry very unlikely.^22^ In this instance, the more FF-looking electrograms go from distal to proximal (yellow circle in **Figure 2**), whereas the first NF (red arrows and circle in **Figure 2**) and second NF (blue arrows and circles in **Figure 2**) signals had 2 opposing activation wavefronts **(Figure 2, Video 1)**. As both components of the DP are sharp NF characteristics (**Figure 2**, blue and red ones) that are associated with WFC sites, we can easily rule out the peri-mitral re-entry as unlikely to form critical sites for ablation in the current case.^22^ Only the single-point pragmatic ablation (red asterisk in **Figure 2**, pink point in **Video 1**) terminated the tachycardia. It surely must be conceded that the activation sequence is only the first step in determining the mechanism, and assessing the relevance of any site is best done by post-pacing interval mapping. However, understanding the nature of complex electrograms is key to discerning the mechanism and likely site of origin of either the focus or vulnerable circuit site of the tachycardia.

## Figures and Tables

**Video 1:** Activation mapping shows the re-entry circuit at the left atrial anteroseptal area; collision of the wavefronts on the mid-distal coronary sinus is seen as compatible with the double potential within the coronary sinus.
